# Wild-Type Transthyretin Amyloid Depositions in the Subcutaneous Fat and Skeletal Muscles of a Nonagenarian Who Had Heart Failure With Preserved Ejection Fraction and No Myocardial Technetium-99m-Labeled Pyrophosphate Uptake

**DOI:** 10.7759/cureus.84759

**Published:** 2025-05-24

**Authors:** Koji Takahashi, Satoshi Yoshida, Mitsuharu Ueda, Shigeki Uemura, Katsuji Inoue

**Affiliations:** 1 Department of Community Emergency Medicine, Ehime University Graduate School of Medicine, Matsuyama, JPN; 2 Department of Dermatology, Ehime University Graduate School of Medicine, Matsuyama, JPN; 3 Neurology, Kumamoto University, Kumamoto, JPN; 4 Department of Cardiology, Yawatahama City General Hospital, Yawatahama, JPN

**Keywords:** bone-avid tracer, extracardiac soft tissue, heart failure, myocardium, wild-type transthyretin amyloidosis

## Abstract

Misfolded amyloid fibrils composed of transthyretin (ATTR) cause ATTR amyloidosis, which is a systemic disease. ATTR amyloidosis can be divided into hereditary and wild-type forms according to the presence or absence of transthyretin *(TTR*) gene mutations. Wild-type ATTR (ATTRwt) amyloidosis, a disease of the elderly, is more prevalent in men. ATTRwt is deposited in many organs and tissues, mainly the heart, lungs, ligaments, and tenosynovium. Orthopedic diseases caused by the deposition of ATTRwt in the ligaments and tenosynovium, such as carpal tunnel syndrome, biceps tendon rupture, spinal canal stenosis, and rotator cuff tears, are known to precede cardiac involvement by several years, leading to ATTRwt cardiomyopathy (ATTR-CM). ATTR-CM can coexist with common heart diseases in patients of heart failure with preserved ejection fraction (HFpEF) and aortic stenosis. Heart failure and rhythm disturbance resulting from ATTR-CM is the leading cause of mortality in patients with ATTRwt. The median life expectancy after the diagnosis of ATTR-CM is low without the administration of disease-modifying drugs. The presence or absence of cardiac involvement is defined by a left ventricular (LV) wall thickness of ≥12 mm on echocardiography, regardless of body size or sex differences. Scintigraphy with bone-avid radiotracers, including technetium-99m-labeled pyrophosphate (Tc-99m-PYP), provides sensitive imaging of myocardial ATTRwt depositions that lead to ATTR-CM. However, there are few reports of histopathologic confirmation of extracardiac ATTRwt deposition prior to myocardial deposition demonstrated by Tc-99m-PYP scintigraphy. We report the case of a 92-year-old woman with acute HFpEF and aortic valve stenosis. Tc-99m-PYP scintigraphy revealed tracer uptake in the subcutaneous fat and skeletal muscles but not in the myocardium. We suspected the patient had ATTR. Biopsy of the subcutaneous abdominal fat with tracer uptake confirmed ATTR deposition. *TTR *gene sequencing revealed no variants, and the results of Tc-99m-PYP scintigraphy led to the diagnosis of ATTRwt with no obvious ATTRwt deposition in the myocardium. Extracardiac Tc-99m-PYP uptake in this case, as in orthopedic diseases due to ATTRwt depositions, reflects a condition prior to myocardial ATTRwt deposition and is considered suspicious for ATTRwt. The clinical significance of reporting this patient is to prompt future studies to test whether ATTRwt is deposited in the hearts of similar patients and whether early detection and treatment of ATTRwt will improve prognosis, which will require the accumulation of similar cases.

## Introduction

In most cases, cardiac amyloidosis is caused by either amyloid fibrils composed of immunoglobulin light chains (AL) or transthyretin amyloid (ATTR), the latter of which can be divided into hereditary and wild-type forms according to the presence or absence of transthyretin (*TTR) *gene mutations [1]. The global epidemiology and mortality rate of hereditary and wild-type ATTR (ATTRwt) amyloidosis has been reported to be heterogeneous [2]. ATTRwt amyloidosis, a systemic disease of the elderly, is characterized by a particularly high prevalence in men. ATTRwt is deposited in many organs and tissues, mainly in the heart, lungs, ligaments, and tenosynovium [3], although organ tropism remains poorly understood [4]. Symptoms of orthopedic diseases due to ATTRwt deposition in soft tissues, such as carpal tunnel syndrome, biceps tendon rupture, spinal canal stenosis, and rotator cuff tears, often precede overt myocardial ATTRwt deposition, leading to cardiomyopathy by 5-15 years [4]. Systemic ATTRwt amyloidosis ultimately results in almost 100% cardiac deposition of amyloid, and ATTRwt cardiomyopathy (ATTR-CM) with heart failure (HF) and arrhythmias is the leading cause of mortality in cases of systemic ATTRwt amyloidosis [1]. The median survival after the diagnosis of ATTR-CM is reported to be approximately five years without the administration of disease-modifying drugs [1,4]. ATTR-CM is no longer considered a rare disease, coexisting in 10% or more of patients with common heart diseases, such as heart failure with preserved ejection fraction (HFpEF) and aortic valve stenosis [5].

The presence or absence of cardiac involvement is defined based on a left ventricular (LV) wall thickness ≥12 mm on echocardiography, regardless of body size or sex differences [6,7]. Female patients with ATTR-CM are generally diagnosed at an older age and have a worse prognosis than male patients. It has recently been reported that this uniform definition of echocardiography is not appropriate for late diagnosis and poor prognosis in female patients [6]. A female patient with ATTR-CM who has an LV wall thickness of 10 mm has been reported [8].

Technetium-99 m-labeled (Tc-99m) pyrophosphate (PYP) and 3,3-diphosphono-1,2-propanedicarboxylic acid (DPD) scintigraphy has a high sensitivity (90-100%) for detecting myocardial ATTRwt depositions before the myocardial wall thickness increases; therefore, it is a useful modality for the early diagnosis of ATTR-CM [7,9,10]. In addition, reports of extracardiac Tc-99m-PYP/DPD uptake in ATTR-CM are increasing [10-12]. However, Tc-99m-PYP/DPD is a bone-avid radiotracer that is taken up by organs and tissues without ATTR deposition [13]. Thus, it is necessary to evaluate the association between scintigraphy and histopathological results in extracardiac tissues and organs with Tc-99m-PYP/DPD uptake, even in patients with myocardial tracer uptake [11,14,15]. Takahashi et al. reported a case of an elderly patient with atrial fibrillation-related HFpEF and aortic stenosis in whom Tc-99m-PYP scintigraphy showed tracer accumulation in the soft tissues, including skeletal muscles and subcutaneous fat, rather than myocardium, and a biopsy of the soft tissue with Tc-99m-PYP uptake revealed ATTRwt deposition [16]. Extracardiac Tc-99m-PYP uptake in this case, as in orthopedic diseases due to ATTRwt depositions, reflects a condition prior to myocardial ATTRwt deposition and is considered suspicious for ATTRwt amyloidosis.

Here, we report the case of a nonagenarian female patient with sinus rhythm and acute decompensated HFpEF complicated by aortic stenosis. The patient was suspected of having cardiac amyloidosis because she had HFpEF (left ventricular ejection fraction (LVEF) >50%) with aortic stenosis, although echocardiography revealed LV wall thickness of <12 mm. In this patient, Tc-99m-PYP scintigraphy revealed tracer uptake in the various skeletal muscles and subcutaneous fat but not in the myocardium. We suspected the patient had ATTR amyloidosis because the pattern of extracardiac tracer accumulation was very similar to that of the previously reported case [16]. Furthermore, no other disease would produce extraosseous tracer accumulation in the skeletal muscles or subcutaneous fat [13]. A biopsy of subcutaneous abdominal fat with Tc-99m-PYP uptake confirmed ATTR deposition, and *TTR *gene sequencing revealed no variants. Thus, the patient was diagnosed with ATTRwt amyloidosis with no obvious ATTRwt deposition in the myocardium. The authors believe that this report will prompt further studies to verify cardiac depositions of ATTRwt in similar patients and determine whether early detection and treatment of ATTRwt amyloidosis will improve the patient's prognosis.

## Case presentation

A 92-year-old Japanese woman (height, 145.0 cm; weight, 44.2 kg; body surface area, 1.29 kg/m^2^) was admitted to our hospital with a four-day history of exertional dyspnea, indicating a New York Heart Association (NYHA) functional class of III. The patient had no history of smoking or alcohol consumption. Her medical history included long-standing hypertension, resection for sigmoid colon cancer performed 13 years prior to presentation, and total knee arthroplasty for left knee osteoarthritis performed 11 years prior to presentation. The patient was prescribed oral azilsartan and amlodipine besilate at another clinic.

In the emergency room, her vital signs were as follows: temperature, 36.7°C; pulse rate, 65 beats per minute; systemic blood pressure, 147/73 mmHg; and oxygen saturation (measured on room air using a pulse oximeter), 91%. Auscultation revealed a grade III/VI systolic ejection murmur at the upper right sternal border and wet rales bilaterally in the lower lung fields. The liver and kidneys were not palpable, but slight pretibial edema was noted. Blood tests revealed hypoalbuminemia with normal liver enzymes, declined renal function with a declined estimated glomerular filtration rate (eGFR) and increased urinary protein-to-creatinine ratio of 0.90 g/gCr, elevated brain natriuretic peptide and high-sensitivity cardiac troponin I levels, and iron deficiency anemia (Table 1). An arterial blood gas analysis on room air indicated mild hypoxemia with hypocapnia due to respiratory compensation. 

**Table 1:** Laboratory test values of the patient upon admission and four months after treatment ALT: alanine aminotransaminase; BNP: brain natriuretic peptide; eGFR: estimated glomerular filtration rate; Fe: serum iron; HCO_3_^-^: bicarbonate ion; hs-cTnI: high-sensitivity cardiac troponin I; PCO_2_: partial pressure of carbon dioxide oxygen; PO_2_: partial pressure of oxygen; TIBC: total iron binding capacity *Determined through arterial blood gas analysis on room air

Parameters	Patient Value		Reference Range
	On admission	Four months after treatment	
Albumin (g/dL)	3.0	2.5	3.8–5.3
ALT (U/L)	15	8	0–31
Creatine kinase (U/L)	101	40	≤165
eGFR (mL/min per 1.73 m^2^)	23	31	90–120
Potassium (mEq/L)	5.2	4.2	3.6–5.0
hs-cTnI (pg/mL)	40.0	47.5	≤18.4
BNP (pg/mL)	3,254	762.3	≤18.4
Glycated hemoglobin A1c (%)	6.2	5.5	4.6–6.2
White blood cells (/μL)	4,800	5,200	4,000–8,500
Red blood cells (/μL)	290 × 10^4^	363 × 10^4^	360 × 10^4^–500 × 10^4^
Hemoglobin (g/dL)	7.9	10.9	12.0–16.0
Platelet (/μL)	22.3 × 10^4^	22.1 × 10^4^	14 × 10^4^–40 × 10^4^
TIBC (μg/dL)	265		220–420
Fe (μg/dL)	18		50–160
Ferritin (ng/mL)	20.3		3.8–123.1
pH	7.453*		7.35–7.45
PCO_2_ (mmHg)	35.0*		35–45
PO_2_ (mmHg)	74.0*		80–100
HCO_3_^-^ (mmol/L)	23.9*		24.2–29.8
Base excess (mmol/L)	0.2*		-2.5–2.5
Lactic acid (mmol/L)	0.69*		0.4–1.4

**Figure 1:**
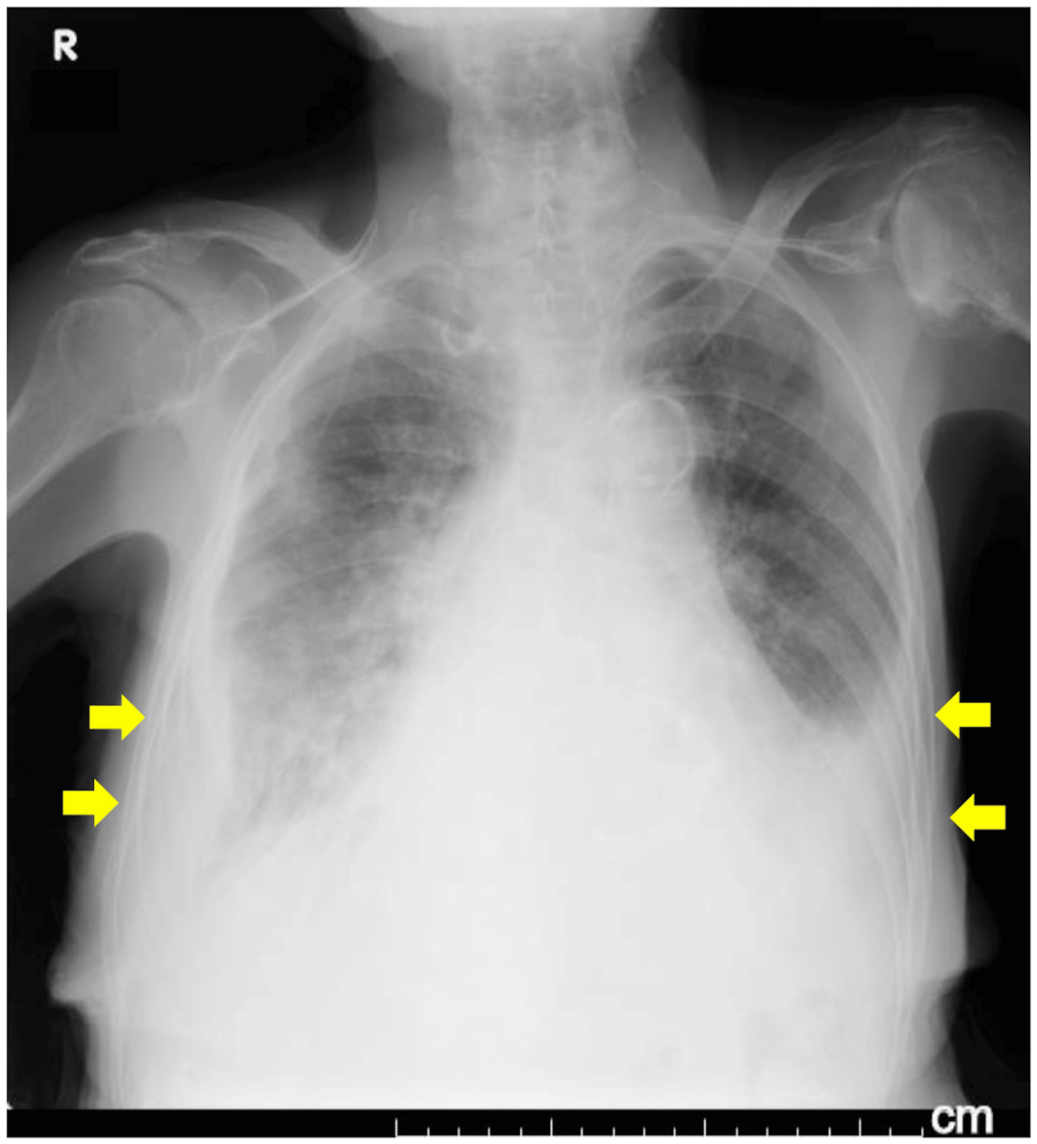
Chest radiography on admission showing cardiomegaly with pulmonary congestion and pleural effusion (arrows) NOTE: Cardiothoracic ratio could not be accurately measured

**Figure 2:**
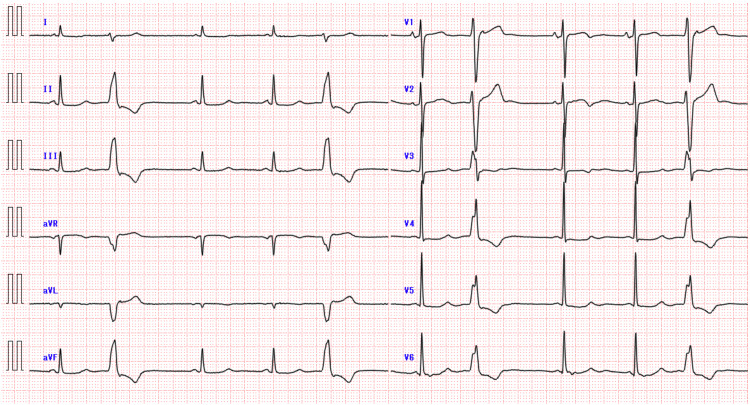
Electrocardiogram on admission showing sinus rhythm with a heart rate of 59 beats/minute, ST–T abnormalities, and premature ventricular contractions.

Chest radiography revealed cardiomegaly, pulmonary congestion, and pleural effusion (Figure 1). Electrocardiography revealed a sinus rhythm with ST-T abnormalities and premature ventricular contractions (Figure 2).

**Figure 3:**
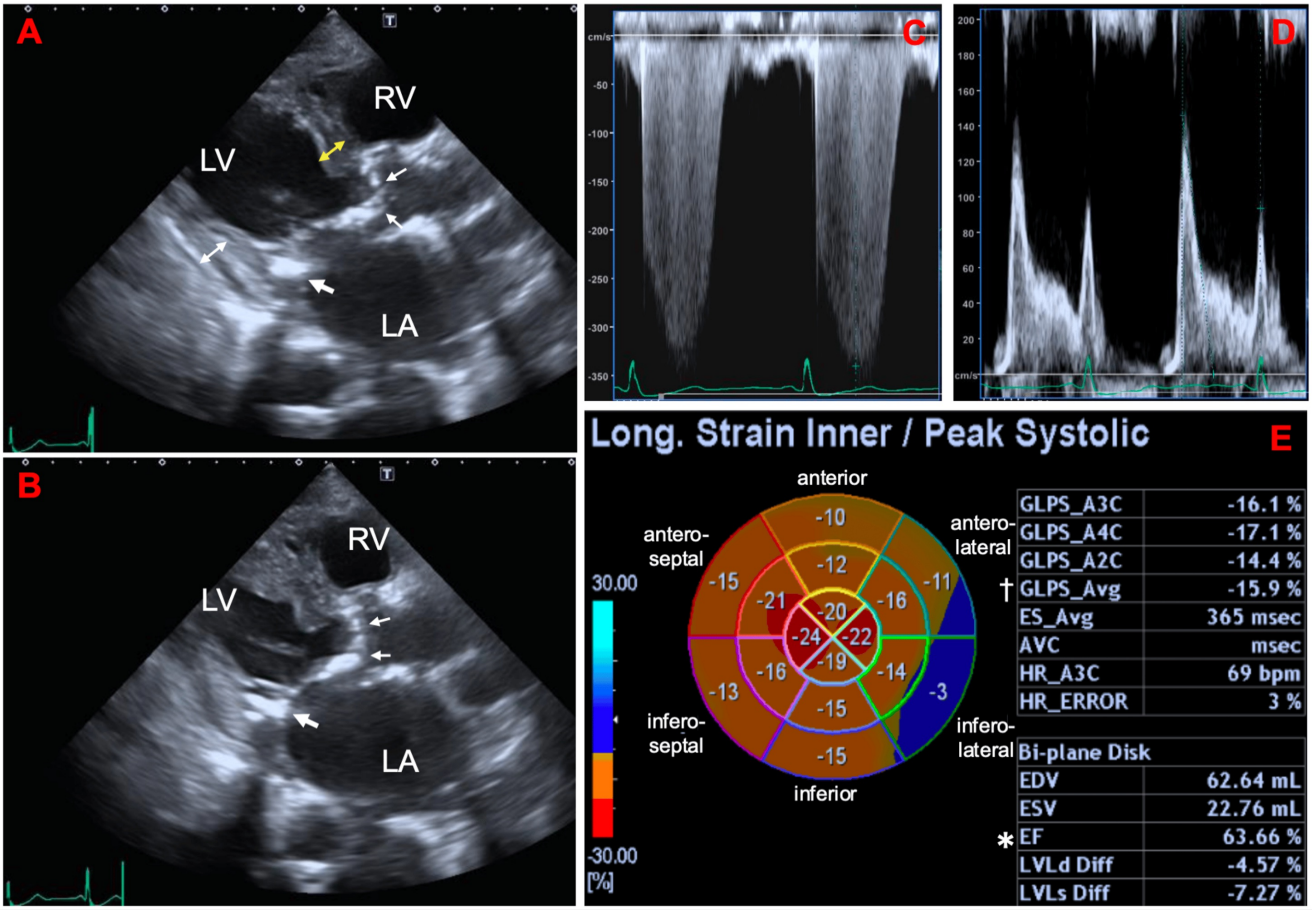
Transthoracic echocardiography on admission (A–D) and on hospital day 8 (E) A parasternal long-axis view at end-diastole (A) and end-systole (B) of transthoracic echocardiography on admission shows normal LV wall motion with a preserved ejection fraction of 66.5%, increased LV wall thickness with an interventricular septum thickness (yellow double arrow) of 10.8 mm, a normal LV posterior wall thickness (white double arrow) of 9.3 mm, a normal end-diastolic LV dimension of 46.8 mm, and an dilated left atrium with a left atrial volume index of 102.6 mL/m^2^. In addition, moderate-to-severe aortic valve stenosis with a peak jet velocity of 3.7 m/sec, a mean/peak transvalvular pressure gradient of 30/54 mmHg, and an aortic valve area of 0.68 cm^2^ is shown (small arrows). The tricuspid regurgitation jet on continuous wave Doppler shows an increased right ventricle-to-right atrial pressure gradient of 47 mmHg (C). Diastolic LV parameters reveal a restrictive pattern with the ratio of mitral peak E-wave velocity to A-wave velocity of 1.56, a deceleration time of the mitral peak E-wave of 160 msec, and an average ratio of mitral peak E-wave to pulsed-wave tissue Doppler-derived mitral annular e’ velocity of 19.7 (D). The bull’s eye map (with the apex at the center of the color-coding map), obtained on hospital day 8 (E) illustrates the segmental longitudinal LV peak systolic strain (LPS) values of the 16-segment model generated by the speckle-tracking analysis of the two-dimensional LV images acquired from apical 2-,3-, and 4-chamber views (A2C, A3C, and A4C, respectively). It shows a mildly deteriorated global LPS of –15.9% (dagger), preserved LVEF of 63.7% (asterisk), and no apical sparing pattern with a relative apical longitudinal strain of 0.79. The large arrow indicates mitral annular calcification. LA: left atrium; LV: left ventricle; RA: right atrium; RV: right ventricle.

Transthoracic echocardiography revealed normal LV wall motion with a preserved ejection fraction (EF) (Video 1), an increased interventricular septum thickness (IVST) of 10.8 mm, a normal LV posterior wall thickness, and a normal end-diastolic LV dimension (Figure 3A, 3B). In addition, moderate to severe aortic valve stenosis, a dilated left atrium, and an increased right ventricle-to-right atrial pressure gradient were present (Figure 3C). Diastolic LV parameters showed a restrictive pattern (Figure 3D).

**Video 1:** Transthoracic echocardiography A parasternal long-axis view of transthoracic echocardiography shows a normal left ventricular wall motion, a mildly increased interventricular septum thickness, a dilated left atrium, a calcified aortic valve with restricted opening motion, and a mitral annular calcification.

Thus, the diagnosis was acute decompensated HFpEF with anemia as a trigger for acute decompensation and moderate-to-severe aortic valve stenosis. Treatment included loop diuretics and sodium-glucose cotransporter-2 inhibitors for acute decompensated HFpEF and iron supplementation for iron deficiency anemia.

**Figure 4:**
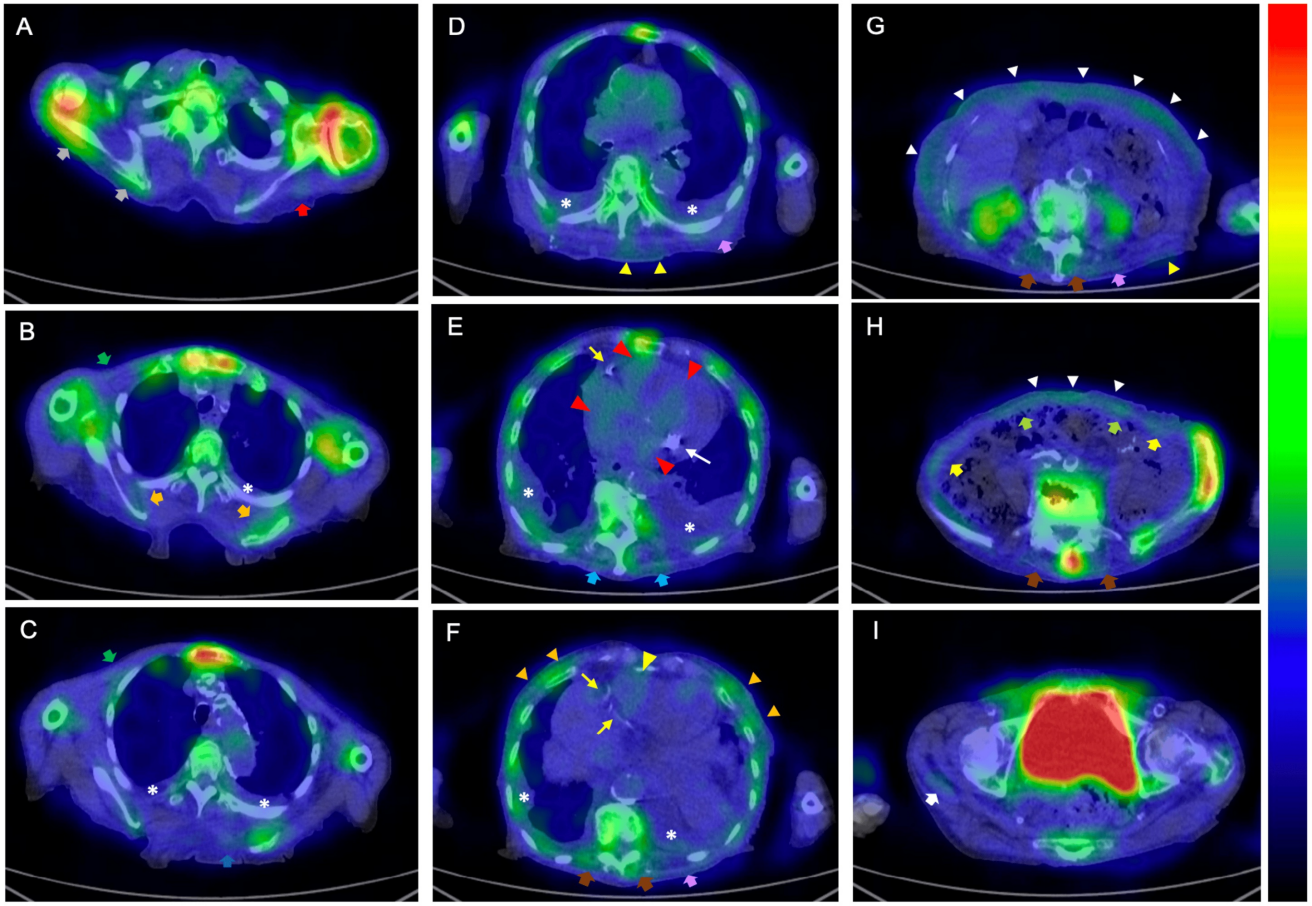
SPECT images of technetium-99m-labeled pyrophosphate (Tc-99m-PYP) scintigram with fusion to CT images obtained 2 hours after injection of the radiotracer Horizontal plane of the (A) shoulder, (B–D) upper chest, (E, F) heart, (G, H) abdomen, and (I) pelvis. In SPECT/CT fusion images displayed in color, Tc-99m-PYP uptake at the sternum is considered the maximum, whereas that at other sites is expressed as relative values. Tc-99m-PYP uptake in the region of interest is considered positive when shown in a color indicating approximately 40% or more of the uptake in the sternum (yellow–green on the color bar) and negative when shown in a color indicating less than 40% (blue). There is no tracer uptake in either the atrial or ventricular myocardium. The red arrowheads indicate cardiac pool tracer uptake. Accumulation of Tc-99m-PYP in the deltoid (red arrow), infraspinatus (gray arrows), subscapularis (orange arrows), pectoralis major (green arrows), rhomboid (blue arrow), latissimus dorsi (purple arrows), trapezius (light blue arrows), erector spinae (brown arrows), abdominal oblique (yellow arrows), rectus abdominis (light green arrows), and gluteus (white arrow) muscles, mammary gland (orange arrowheads), subcutaneous back (yellow arrowheads), and subcutaneous abdominal fat (white arrowheads) is shown. Stars, thin yellow arrows, and thin white arrows indicate pleural effusion, calcification of the coronary arteries, and mitral annular calcification, respectively. SPECT: Single photon emission computed tomography

Follow-up echocardiography performed on the hospital day 8 showed a mildly deteriorated global longitudinal strain and no apical sparing pattern (Figure 3E). Although the LV wall thickness did not exceed 12 mm, Tc-99m-PYP scintigraphy was performed because she was an elderly female patient with HFpEF accompanied by aortic valve stenosis [5,6]. Two hours after intravenously injecting the radiotracer (20 mCi), chest-centered and abdomen-centered Tc-99m-PYP scintigraphy images were obtained using a dual-head single-photon emission computed tomography (SPECT) camera equipped with low-energy high-resolution collimators [11,12]. Immediately after obtaining the SPECT images, low-dose CT scans were acquired separately using a dedicated CT scanner during an end-expiratory breath-hold for the fusion of SPECT and CT images. No myocardial Tc-99m-PYP uptake was observed; however, uptake was observed in the extracardiac soft tissues, including the subcutaneous fat and various skeletal trunk muscles (Figure 4).

**Figure 5:**
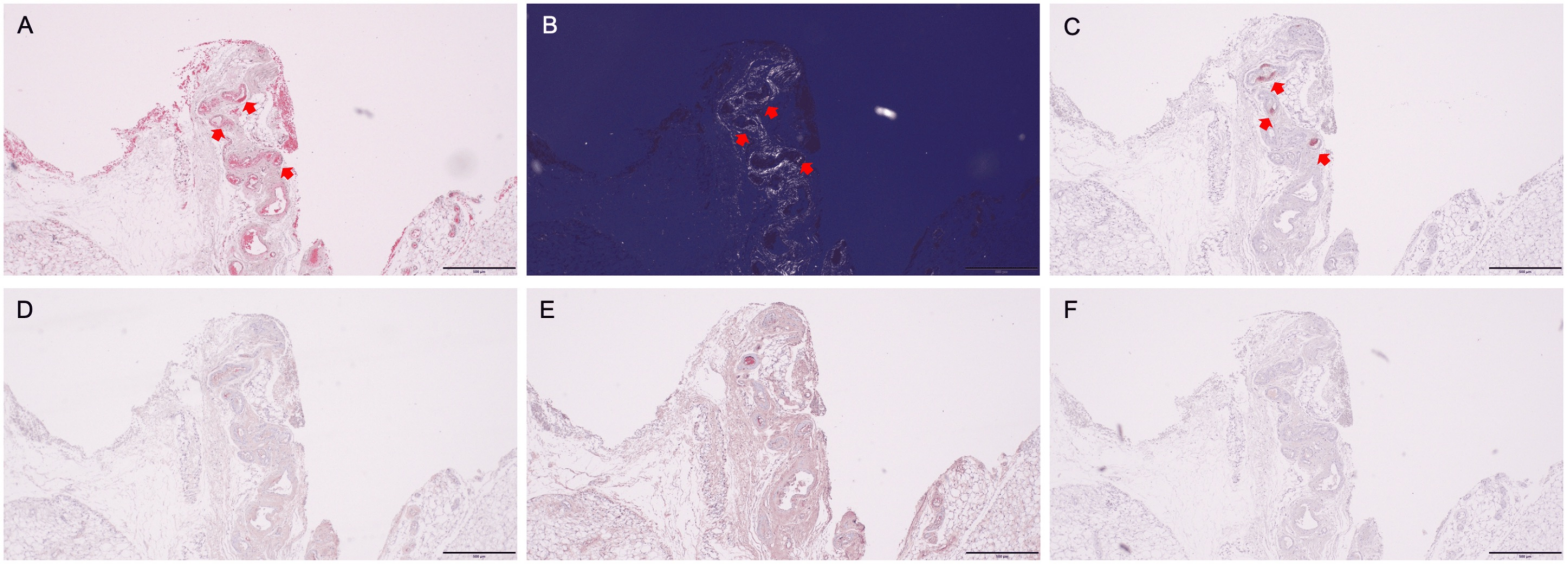
Histopathological images of specimens obtained from the subcutaneous abdominal fat biopsy Congo red staining performed at Kumamoto University revealed amyloid deposits showing red–orange under a light microscope (A), and apple–green birefringence under a cross-polarized light microscope (B) (arrows). Immunohistochemical staining subsequently performed at Kumamoto University using a panel of type‐speciﬁc antibodies against the most common amyloidogenic proteins causing cardiac amyloidosis, including anti‐transthyretin 115–124 (polyclonal rabbit anti-human prealbumin, custom made) (C), anti‐kappa light chain 116–133 (polyclonal rabbit anti‐human kappa light chains, custom made) (D), anti‐lambda light chain 118–134 (polyclonal rabbit anti‐human lambda light chains, custom made) (E), and anti‐amyloid A (monoclonal mouse anti-human amyloid A, DAKO) (F), shows that the antibody raised against the anti‐transthyretin 115–124 is positive (C: arrows), and the antibodies raised against other amyloid proteins are negative (D–F). The scale bars in all panels indicate 500 μm.

We suspected the patient had ATTR amyloidosis because the pattern of extracardiac tracer accumulation was very similar to that of a previously reported case [16]. Furthermore, no other disease could produce extraosseous tracer accumulation in the skeletal muscles or subcutaneous fat [13]. A biopsy of the subcutaneous abdominal fat with tracer uptake confirmed ATTR deposition (Figure 5).

*TTR *gene sequencing revealed no variants. Therefore, the patient was diagnosed with ATTRwt amyloidosis without obvious myocardial ATTR deposition, which was considered a bystander in acute HF, although cardiac magnetic resonance (CMR) imaging and endomyocardial biopsy were not performed.

The patient continued to be prescribed diuretics and sodium-glucose cotransporter-2 inhibitors for HFpEF. There was no indication for prescribing disease-modifying drugs, such as transthyretin tetramer kinetic stabilizers for ATTRwt amyloidosis, because of no apparent myocardial amyloid deposits. Four months after starting treatment, the patient’s heart failure was NYHA functional class II with a prescription of 15 mg azosemide daily and 10 mg dapagliflozin propylene glycolate hydrate daily. Blood test results are shown in Table 1.

## Discussion

This was a case of an elderly patient with HFpEF and aortic stenosis in whom Tc-99m-PYP scintigraphy proved useful in imaging extracardiac ATTRwt deposition prior to myocardial deposition. The patient was considered to have systemic ATTRwt amyloidosis at the preliminary stage of cardiac amyloidosis.

A multimodality assessment is needed to diagnose and monitor ATTR-CM [17]. Electrocardiography and echocardiography are the first-line imaging modalities in most patients; however, those diagnostic tools are less helpful in the early course of cardiac amyloidosis [7,18]. Once cardiac amyloidosis is suspected, Tc-99m-PYP/DPD scintigraphy has become an essential diagnostic test for detecting myocardial ATTRwt depositions before the myocardial wall thickness increases [7]. If tracer accumulation in the myocardium is present and plasma cell dyscrasia leading to AL amyloidosis is excluded by appropriate blood and urine tests, the diagnosis of ATTR-CM can be made without the histopathological detection of ATTR [1]. However, Tc-99m-PYP/DPD scintigraphy rarely shows false negative results [8]. If ATTR amyloidosis is strongly suspected, even in the setting of no myocardial Tc-99m-PYP uptake, other modalities, such as CMR imaging, should be considered. CMR using native T1 mapping and extracellular volume mapping can image tissue characterization corresponding to the burden of myocardial ATTR even in the early stage of ATTR-CM [7,18]. In particular, native T1 mapping, which can be obtained without the use of gadolinium-based contrast media, may be useful in older patients, many of whom have reduced renal function. However, T1 mapping with or without contrast media is not done in our hospital. In addition, we are hesitant to use gadolinium-based contrast agents in patients with impaired renal function. In the current patient, the radiotracer was taken up by soft tissues, and ATTRwt deposition was confirmed by biopsy of the subcutaneous abdominal fat with tracer uptake. Thus, we assumed that Tc-99m-PYP scintigraphy showed a true-negative result for ATTRwt deposition in the myocardium, where there was no tracer uptake, and that no myocardial tracer uptake indicated no or minimal ATTRwt deposition below the sensitivity of scintigraphy. Therefore, systemic ATTRwt amyloidosis was thought to be a bystander in the acute decompensated HFpEF in our patient.

Although the median survival after diagnosis of ATTR-CM is reported to be approximately five years without the administration of disease-modifying drugs [1,4], early-stage ATTR-CM is not considered to have a poor prognosis, even without disease-modifying therapy [19,20]. In addition, treatment with transthyretin tetramer kinetic stabilizers was associated with an improved prognosis in patients with ATTR-CM without heart failure symptoms. However, the heart failure symptoms, in this case, were of HFpEF origin, and ATTRwt amyloidosis without cardiac involvement was considered a bystander to HEpEF. In patients with HFpEF, the survival rate at the five-year follow-up is <40% [21]. It is unclear whether ATTRwt will begin to accumulate in our patient’s myocardium in the future and, if so, when. In the current patient, HFpEF developed in the setting of no or small myocardial ATTRwt deposition, although anemia triggered the acute exacerbation of HF. This may make it difficult to manage HF due to further ATTRwt deposition in the myocardium. Thus far, there are no reports on the results of disease-modifying therapies for ATTRwt amyloidosis without cardiac involvement, as in our case. Therefore, more patients with heart disease, such as HFpEF, in the setting of ATTRwt amyloidosis without cardiac involvement, are required to verify whether amyloid will be deposited in the heart in the near future and whether the early detection of cardiac ATTRwt involvement and early treatment with disease-modifying agents could improve symptoms and mortality. According to Japanese guidelines, tafamidis is indicated for patients with an interventricular septal thickness (IVST) >12 mm and heart failure symptoms [22]. Therefore, at this time, there is no indication for the administration of a disease-modifying agent for systemic ATTRwt amyloidosis without documented cardiac amyloid deposition, as in our case.

When performing Tc-99m-PYP scintigraphy, the SPECT image must be superimposed on the CT image obtained immediately after scintigraphy to accurately identify the site of tracer accumulation because SPECT has a low spatial resolution [18]. At our hospital, Tc-99m-PYP and CT were performed using separate scanners, and care was taken to maintain the same posture during SPECT and CT [11,12]. As shown in Figure 4, there was some misregistration or blurring of the SPECT/CT fusion images, mainly due to patient movement and slightly different postures resulting from using separate SPECT and CT scanners. In addition, the partial volume effect inherent in SPECT imaging, which is caused by the limited resolution of the SPECT scanner, leads to a blurred image limited by the accuracy of the regional radiotracer uptake. In particular, the marked tracer uptake in the bone and bladder made it difficult to assess the presence or absence of soft tissue uptake in their vicinity. Moreover, the localization and separation of the tracer taken up in the thin subcutaneous abdominal fat from that in the rectus abdominis muscle was difficult because of misregistration and partial volume effects (Figure 4H). At our hospital, abdomen-centered images and chest-centered images are obtained to assess the ATTR burden and decide the biopsy sites other than endomyocardial biopsy [15,23].

Regarding how to manage patients with the same condition as our patient, we first treat HFpEF and monitor disease progression of moderate to severe aortic stenosis and ATTRwt amyloidosis without cardiac involvement. In particular, we adhere to the proposed follow-up scheme in patients with ATTR-CM, which includes tracking biomarkers, such as natriuretic peptide and cardiac troponin levels, and performing electrocardiograms and echocardiograms every six months to one year [1]. If ATTRwt amyloidosis without cardiac involvement is suspected to develop into cardiac amyloidosis, as indicated by the criteria for disease progression [24], Tc-99m-PYP scintigraphy should be repeated. In our case, the estimated glomerular filtration rate and plasma natriuretic peptide values were abnormal, even without cardiac amyloid deposition. Therefore, a staging system, such as the National Amyloidosis Center's ATTR staging system, which uses these parameters, will be unable to classify our patient's condition immediately after developing ATTR-CM as an early stage of the disease [19].

## Conclusions

Our patient was diagnosed with ATTRwt amyloidosis at an early stage before myocardial ATTRwt deposition using extracardiac Tc-99m-PYP uptake. Recognition of this extracardiac red flag in ATTR-CM may be important for the early diagnosis of ATTRwt amyloidosis, leading to improved patient outcomes. Our patient should be followed up carefully for future ATTRwt accumulation in the heart, although it is necessary to collect cases in which Tc-99m-PYP accumulates in the skeletal trunk muscles and subcutaneous fat but not in the myocardium, and to investigate their subsequent course.
